# Base composition is the primary factor responsible for the variation of amino acid usage in zebra finch (*Taeniopygia guttata*)

**DOI:** 10.1371/journal.pone.0204796

**Published:** 2018-12-05

**Authors:** Yousheng Rao, Zhangfeng Wang, Wen Luo, Wentao Sheng, Rendian Zhang, Xuewen Chai

**Affiliations:** 1 Department of Biological Technology, Nanchang Normal University, Nanchang, Jiangxi, China; 2 Jiang Xi Province Key Lab of Genetic Improvement of Indigenous Chicken Breeds, Nanchang, Jiangxi, China); Fred Hutchinson Cancer Research Center, UNITED STATES

## Abstract

In the present study, we carried out an examination of the amino acid usage in the zebra finch (*Taeniopygia guttata*) proteome. We found that tRNA abundance, base composition, hydrophobicity and aromaticity, protein second structure, cysteine residue (Cys) content and protein molecular weight had significant impact on the amino acid usage of the zebra finch. The above factors explained the total variability of 22.85%, 25.37%, 10.91%, 5.06%, 4.21%, and 3.14%, respectively. Altogether, approximately 70% of the total variability in zebra finch could be explained by such factors. Comparison of the amino acid usage between zebra finch, chicken (*Gallus gallus*) and human (*Homo sapiens*) suggested that the average frequency of various amino acid usage is generally consistent among them. Correspondence analysis indicated that base composition was the primary factor affecting the amino acid usage in zebra finch. This trend was different from chicken, but similar to human. Other factors affecting the amino acid usage in zebra finch, such as isochore structure, protein second structure, Cys frequency and protein molecular weight also showed the similar trends with human. We do not know whether the similar amino acid usage trend between human and zebra finch is related to the distinctive neural and behavioral traits, but it is worth studying in depth.

## Introduction

Amino acids are utilized with different frequencies in various proteins and organisms. Such biases in amino acid usage have been demonstrated extensively in prokaryote and eukaryote genomes, and likely reflect a balance or near balance between the action of mutation, selection, and genetic drift [1–3]. Base composition in a number of species has been shown to correlate with the amino acid content of proteins. This trend has been attributed to the neutral processes or mutation[1, 4–11]. Using a measure based on tRNA-gene copy numbers as a rough estimate of tRNA abundance, a positive correlation between tRNA abundance and the amino acid content has been documented in many organisms, suggesting selection plays an important role in shaping amino acid frequencies[3, 12–17]. In addition, intragenomic analyses have suggested that factors like hydrophobicity, aromaticity, cysteine residue (Cys) content, gene function, metabolic cost, mean molecular weight and gene expression level also have significant impact on the global amino acid composition of each species[1–3, 18–23].

Although there are many influencing factors, the base composition was considered as the driving force in the amino acid usage. Knight et al. [1] made a comparative study on the impact of GC content on codon usage and amino acid usage for bacteria, archaea and eukaryotes with limited gene sample. They concluded that amino acid responses were determined by the mean GC content of their codons (explaining 71–79% of the variance). However, Rao et al. [3] reported that only approximately 10–40% variation of amino acid usage could be explained by GC content in chicken. A recent study argued that the impact could be also in the opposite direction, i.e. the selection at the amino acid level could affect the nucleotide content and codon usage significantly [24].

In the avian group, Rao et al. [3] made a systematic study of the amino acid usage in the chicken proteome. They found that the relative amino acid usage was strongly correlated with the tRNA abundance. Correspondence analysis also suggested that the main factors responsible for the variation of amino acid usage in chicken were hydrophobicity, aromaticity, and genomic GC content. In the present study, we carried out an examination of the amino acid usage in the zebra finch (*Taeniopygia guttata*) proteome. The aim of this study is to explore which are the main parameters that shape the global amino acid usage in the zebra finch, to assess the similarities and differences between the two bird genomes, and to describe their biological implications.

## Materials and methods

### Sequence data

In this study, gene sequences, coding DNA sequences (CDSs), or complete mRNA sequences corresponding to all annotated genes in *Taeniopygia guttata* genome were downloaded from Ensembl. For this data collection, a strict criteria was defined: (1) Only nuclear genes with known protein products (rather than a novel or predicted transcripts) were included; (2) Only genes with complete CDSs were included; (3) Genes with a CDS that did not begin with an ATG start codon, or did not have a length ≥ 300 bp, or did not occur in multiples of three nucleotides, or contained an internal stop codon, were discarded. We declare that all the data used in this study are public.

### tRNA gene copy number data

The copy numbers of individual tRNA genes in the *Taeniopygia guttata* genome were taken from (http://gtrnadb.ucsc.edu/GtRNAdb2/genomes/eukaryota/Tgutt2/). In this data set, pseudogenes have already been removed.

### Correspondence analysis

Correspondence analysis (COA) implemented by CodonW 1.4.2 was used to identify the major factors that shape variation in amino acid usage among proteins of *Taeniopygia guttata*. For each gene, the relative amino acid usage (RAAU), the GC content of the CDS (GC_cds_), the GC content at the first, the second and the third position (GC1, GC2 and GC3), the average hydrophobicity (general average hydrophobicity, GRAVY) and the average aromaticity (average aromaticity, Aromo), were calculated by CodonW 1.4.2. We also performed a principal-components analysis (PCA) for the genes of zebra finch. The results were similar to the correspondence analysis.

### Statistical analysis

Correlation analysis between variables was performed by SAS Proprietary Software Release 8.1. In order to assess the actual strength of correlation, all correlation coefficients reported in this study were tested independently, excluding the influence of other related variables. To determine the variables contributing to the variability and how they may interact, we performed multiple linear regressions with the variables, excluding those not contributing significantly through the use of the t-statistical logarithm with backward stepwise regression. The significance tests were corrected for multiple testing by the Bonferroni step-down correction [25].

## Results

### Relationship between amino acid usage and tRNA gene copy number

**Fig 1:**
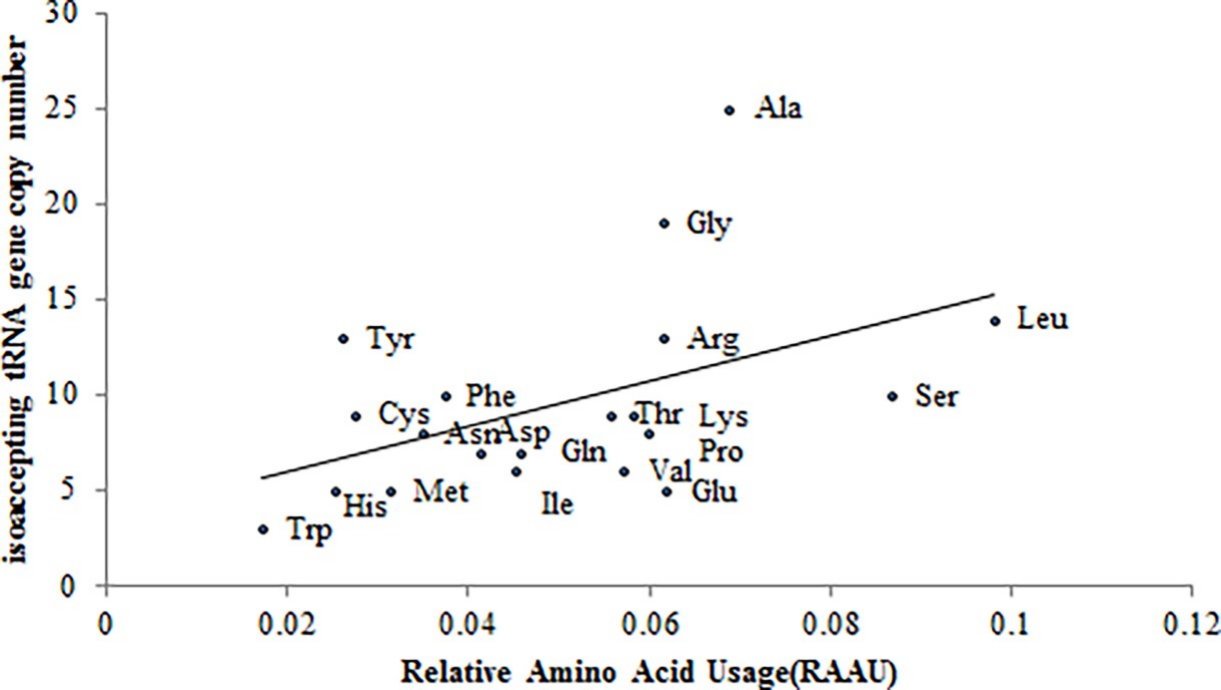
Relationship between the relative amino acid usage and the isoaccepting tRNA gene copy number. The tRNA gene copy numbers for each codon in the *Taeniopygia guttata* genome was taken from http://gtrnadb.ucsc.edu/GtRNAdb2/genomes/eukaryota/Tgutt2/ (August 2, 2017). The isoaccepting tRNA gene number was summed for each amino acid. The relative amino acid usage (RAAU) for each amino acid was calculated by CodonW 1.4.2. The average RAAU values of amino acid was correlated with the isoaccepting tRNA gene copy numbers significantly (r = 0.478, p = 0.038).

The relative amino acid usage (RAAU) for each gene was calculated by CodonW 1.4.2. We found that the amino acids were not equally used in the zebra finch proteome. The average RAAU of Leu, Ser, Ala, Lys, Glu and Arg was relatively high (> 6%); otherwise, some amino acid RAAU such as Cys, His, Trp, Tyr was relatively low (< 3%). We retrieved the tRNA gene copy numbers for each codon in the *Taeniopygia guttata* genome. The isoaccepting tRNA genes were summed for each amino acid. Our data demonstrated that the average RAAU was correlated with the isoaccepting tRNA gene copy numbers significantly (r = 0.478, p = 0.038) (Fig 1).

### Factorial correspondence analysis for amino acid usage

**Fig 2:**
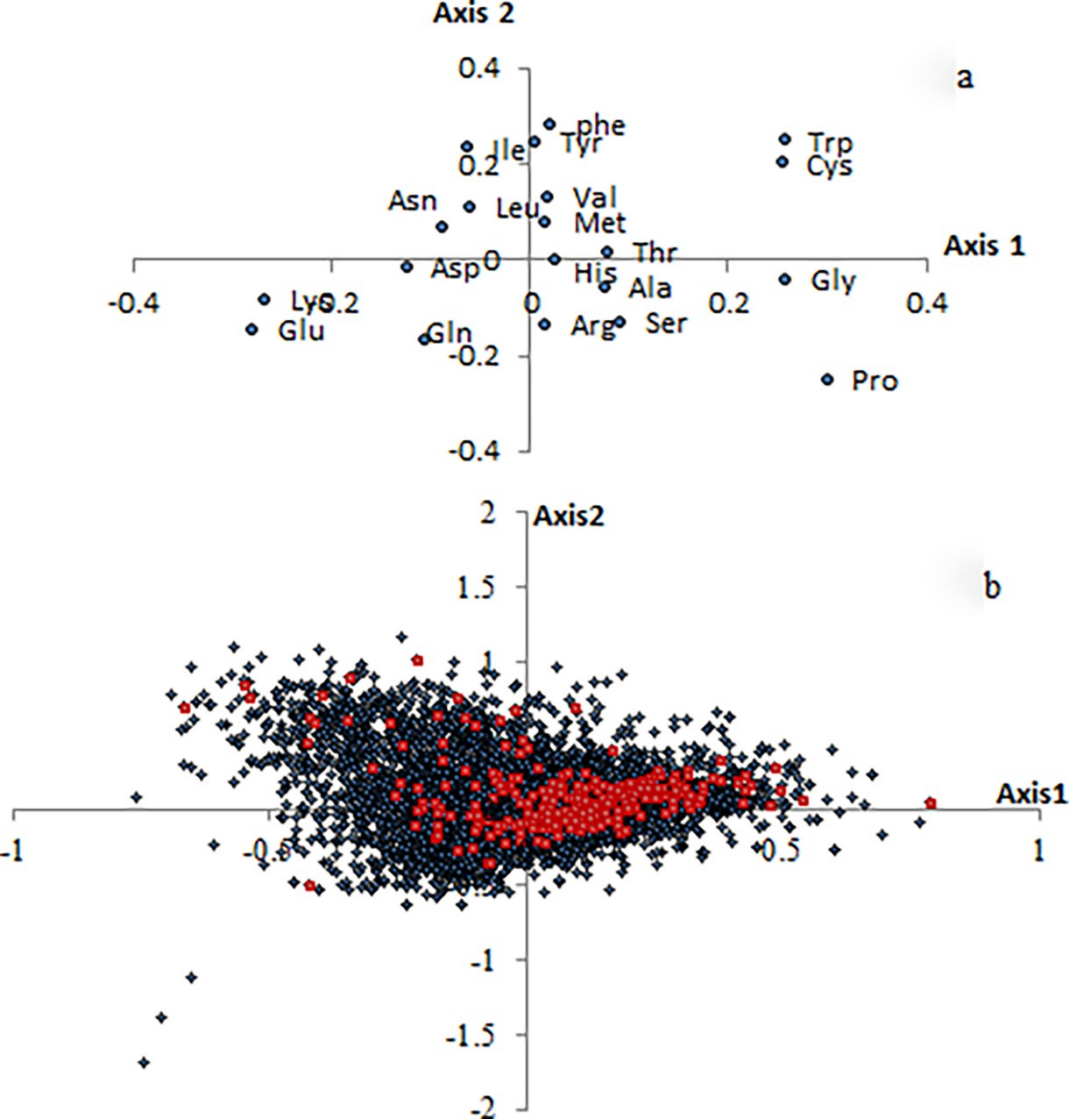
Distribution of the amino acids and genes on the first two axes of the correspondence analysis. a. Representation of the first two axes of the correspondence analysis performed on the amino acid frequency of *Taeniopygia guttata* protein. b. Representation of the first two axes of the correspondence analysis performed on the amino acid frequencies of 8109 *Taeniopygia guttata* genes. Membrane proteins are indicated by red dots. The total number of membrane proteins was 298. The percentage of membrane proteins with the positive value account for 72%.

Correspondence analysis (COA) was used to explore the major factors shaping variation in amino acid usage among *Taeniopygia guttata* proteins. The coordinate of each gene on each axis and the fraction of the total variation accounted for by each axis was generated by COA. Our data indicated that 4 of the 19 axes accounted for almost 50% of the total variance (47.79%) in amino acid composition of *Taeniopygia guttata* proteins. The first major axis accounted for 17.34% of the total variance, and the 2nd, 3rd, 4th major axis accounted for 14.68%, 8.5%, 7.27% of the total variance, respectively. The distribution of the amino acid residues and the total genes for the first two axes were shown in Fig 2.

### Impact of GC content on amino acid usage

**Fig 3:**
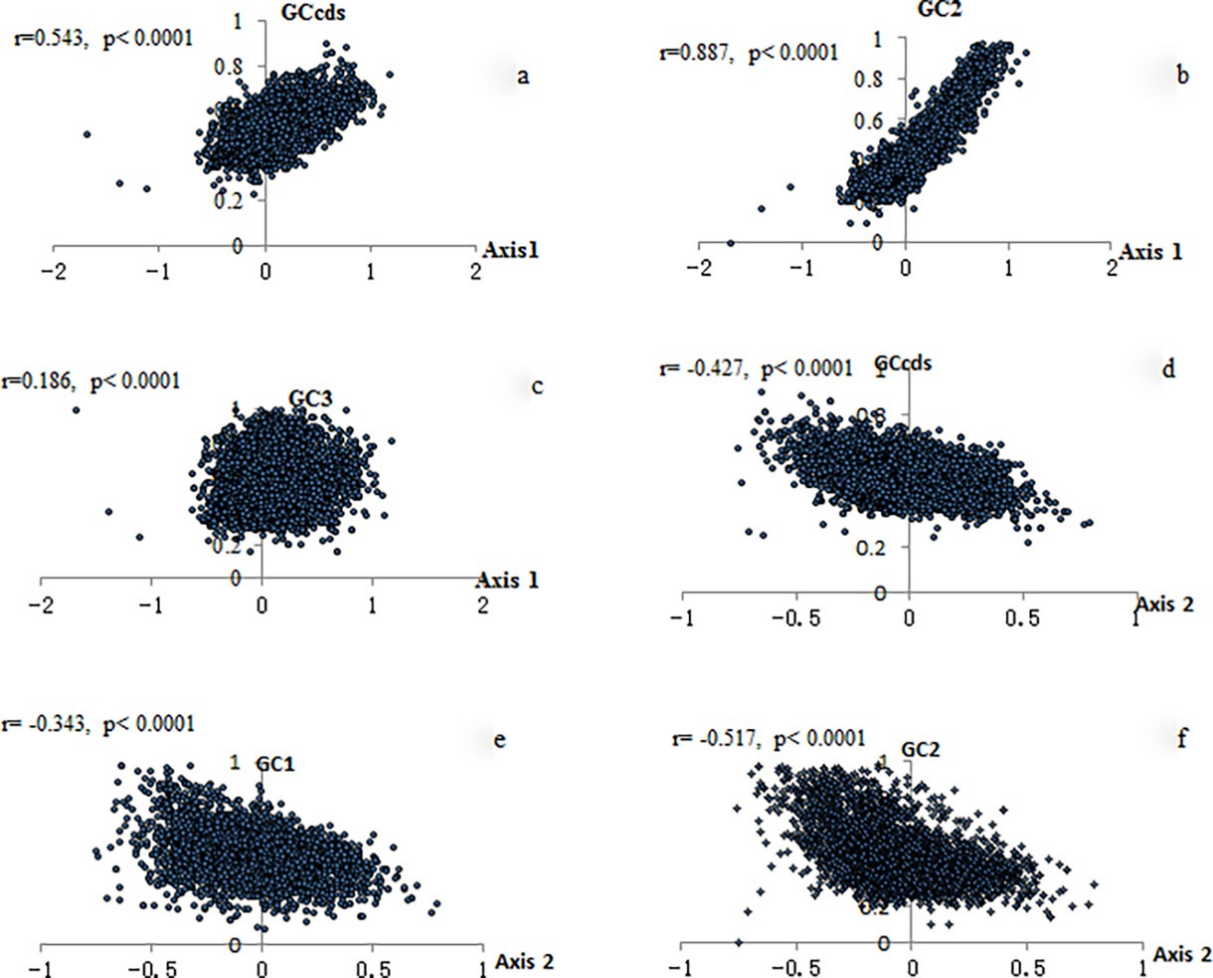
Relationship between GC content and axis 1,axis 2. a. Axis 1 positively correlated with GC_cds_ significantly. b. Axis 1 strongly correlated with GC2 positively. c. Axis 1 weakly correlated with GC3 positively. d. Axis 2 negatively correlated with GCcds significantly. e. Axis 2 negatively correlated with GC1 significantly. f. Axis 2 negatively correlated with GC2 significantly.

The GC content of the CDS (GC_cds_), the GC content at the first, the second and the third position (GC1, GC2 and GC3), were calculated by codonW 1.4.2. As shown in Fig 3, axis 1 was positively correlated with the GC_cds_, GC2, and GC3, significantly (axis 1 vs. GC_cds_, r = 0.543, p < 0.0001; axis 1 vs. GC2, r = 0.887, p < 0.0001; axis 1 vs. GC3, r = 0.186, p < 0.0001). Multiple regression analysis indicated that the main factor was GC2 (R^2^ = 0.788, p < 0.0001). Axis 2 was negatively correlated with GC_cds_, GC1, and GC2, significantly (axis 2 vs. GC_cds_, r = - 0.427, p < 0.0001; axis 2 vs. GC1, r = - 0.343, p < 0.0001; axis 2 vs. GC2, r = - 0.517, p < 0.0001). Multiple regression analysis indicated that the main factors were GC2 and GC1 (R^2^ = 0.425, p < 0.0001). Axis 3 and axis 4 also correlated with the GC content. The main factors for Axis 3 were GC1 and GC2 (R^2^ = 0.556, p < 0.0001), while the main factors affecting Axis 4 were GC1 and GC_cds_ (R^2^ = 0.126, p < 0.0001). According to Sabbía *et al*. [20], we used the GC content of the surrounding regions of gene (25 kb upstream of the initiation codon plus the 25 kb downstream of the stop codon) as an estimator for isochore structure, and made correlation analysis between the estimator and axis 1, axis 2. We found that both axis 1 and axis 2 were significantly correlated with the estimator, suggesting that the isochore structure had a significant impact on the amimo acid usage in zebra finch (axis 1 vs. estimator, r = 0.198, p < 0.0001; axis 2 vs. estimator, r = - 0.16, p < 0.0001).

### Impact of hydrophobicity and aromaticity on amino acid usage

**Fig 4:**
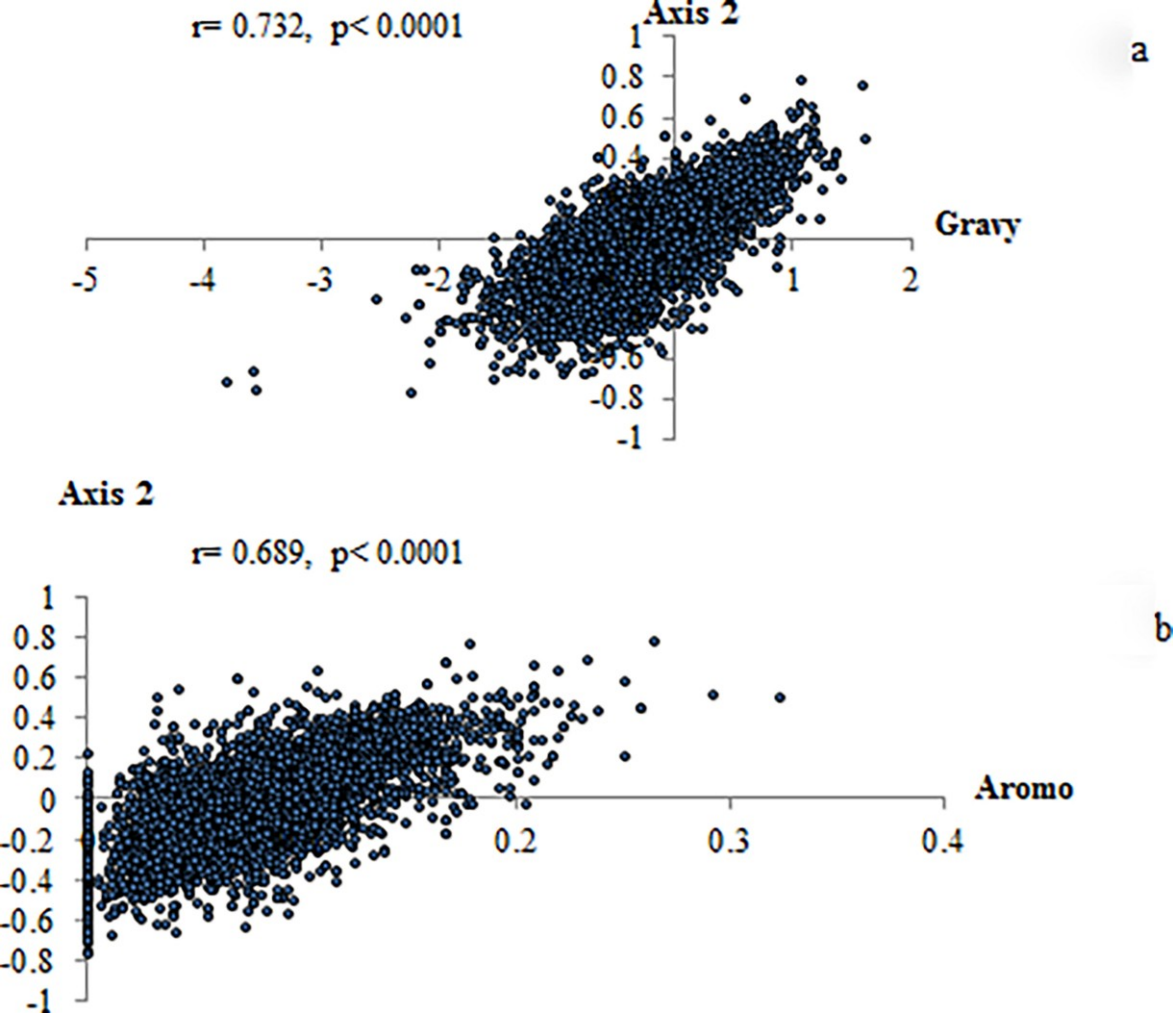
Relationship between axis 2 and the GRAVY score of proteins, the Aromo score of proteins. a. Axis 2 strongly correlated with the GRAVY score of proteins. b. Axis 2 strongly correlated with the Aromo score of proteins.

The average hydrophobicity (general average hydrophobicity, GRAVY) and aromaticity (average aromaticity, Aromo), were calculated by CodonW 1.4.2. As shown in Fig 4, axis 2 was strongly correlated with the GRAVY score of proteins (r = 0.732, p < 0.0001), and the Aromo score of proteins (r = 0.689, p < 0.0001). As axis 2 was also found to be correlated with GCcds, GC1, and GC2 significantly, we made a multiple regression analysis between axis 2 and all 5 variables. Our data indicated that 90.4% of the total variation of axis 2 could be explained by GRAVY, Aromo, GC2 and GC1 (R^2^ = 0.904, p < 0.0001), and 74.3% could be explained by Gravy and Aromo (R^2^ = 0.743, p < 0.0001). Axis 1 also showed a significant correlation with the GRAVY score (r = 0.228, p < 0.0001), but did not correlate with the Aromo score (r = 0.01, p = 0.334). As shown in Fig 2A, the strong hydrophobic amino acid Ile, Val, Phe, Leu, Met, and the aromatic amino acid Tyr, Phe, and Trp, were at the above of the plane (positive values for axis 2). The distribution of genes in Fig 2B indicated that the membrane proteins were related to the distribution of axis 2, in which the majority of them showed a positive value over the axis 2.

### Impact of protein second structure, molecular weight and Cys frequency on amino acid usage

The amount of secondary structure for each protein was predicted by the use of PHD software. The distribution of alpha helix, extend strand, and random coil over the entire protein data set were analyzed. Correlation analyses indicated that extend strand was positively correlated with axis1 (r = 0.157, p < 0.0001) and axis2 (r = 0.354, p < 0.0001). Random coil was positively correlated with axis1 (r = 0.204, p < 0.0001), negatively correlated with axis2 (r = -0.229, p < 0.0001) and axis4 (r = -0.335, p < 0.0001). The second structure could explain 5.06% of the total variability found in our proteins.

Previous studies demonstrated that the molecular weight of proteins had a significant effect on the amino acid usage. The same trend was also found in *Taeniopygia guttata* proteome. The molecular weight of proteins were negatively correlated with axis 1 (r = -0.4261, p < 0.0001). We also analyzed the influence of the Cys frequency on the total variability. We found that Cys frequency was positively correlated with axis 1(r = 0.35, p< 0.0001) and axis 3 (r = 0.494, p < 0.0001). 4.21% of the total variability could be explained by Cys frequency. The CDS length also showed a significant correlation with axis 2 (r = -0.2179, p < 0.0001) and axis 3 (r = -0.1252, p < 0.0001), but with very low coefficients.

## Discussion

In the present study, we carried out a genome scale analysis of the amino acid usage in the zebra finch (*Taeniopygia guttata*). The effects of tRNA abundance, base composition, hydrophobicity and aromaticity, protein second structure, Cys frequency and protein molecular weight on amino acid usage were investigated in detail. We found that the above factors influenced the variability of amino acidic composition of the zebra finch proteome, explaining 22.85%, 25.37%, 10.91%, 5.06%, 4.21%, and 3.14% of the total variability, respectively. Altogether, approximately 70% (71.54%) of the total variability in the *Taeniopygia guttata* proteome could be explained by such factors.

**Fig 5:**
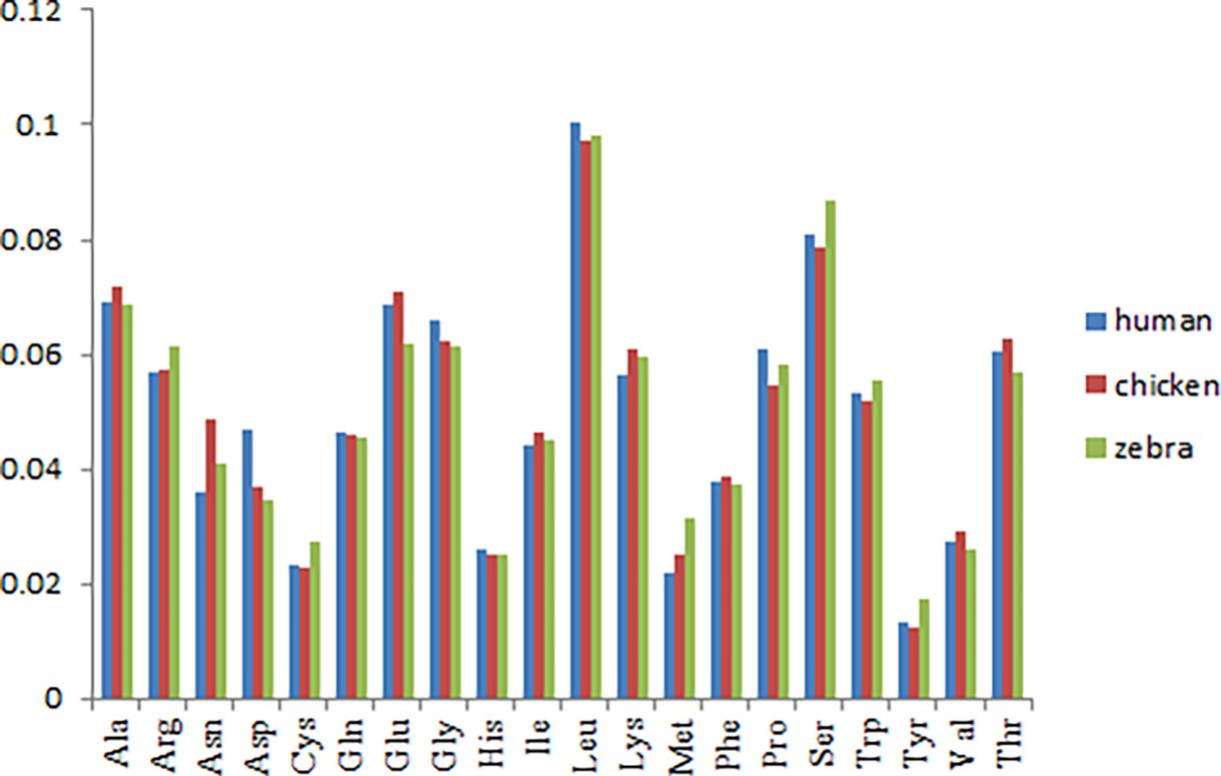
Comparison of the average frequency of various amino acids usage among zebra finch (*Taeniopygia guttata*), chicken (*Gallus gallus*) and human (*Homo sapiens*).

Among the avian species, chicken (*Gallus gallus*) is the best studied representative. The chicken and zebra finch lineages diverged about 100 million years ago. Their genome structures are similar, such as smaller, tighter, marked reduction of interspersed repeats etc., but they differ in many intrachromosomal rearrangements, lineage-specific gene family expansions, the number of long-terminal-repeat-based retrotransposons, and so on [26]. The zebra finch is an ideal model for study on the brain development, as it communicates through learned vocalizations, an ability documented only in human (*Homo sapiens*) and a few other animals, but lacking in chicken [27]. Here, we made a comparison of the amino acid usage among zebra finch, chicken and human. As shown in Fig 5, there was no significant difference in the average use of 20 amino acids. The trends of the various amino acid usage frequency were generally consistent. For example, the contents of Leu, Ser, Ala and Glu were relatively high, otherwise, some amino acid contents such as Cys, His, Met, Tyr were relatively low (S1 Table). Rao et al. [3] argued that the primary factors responsible for the variation of amino acid usage in chicken were hydrophobicity and aromaticity. In that study, axis 1 was strongly correlated with the GRAVY score and Aromo score. This correlation trend was not consistent with the present study. Correspondence analysis and multiple linear regression analysis in the zebra finch indicated that axis 1 was mainly influenced by GC2 (R^2^ = 0.788, p < 0.0001), otherwise, hydrophobicity and aromaticity were main factors impacted on axis 2 (R^2^ = 0.743, p < 0.0001). In other words, base composition was the primary factor responsible for the variation of amino acid usage in zebra finch. This trend is similar to a previous study in human [20]. Other factors affecting the amino acid usage in zebra finch, such as isochore structure, protein second structure, Cys frequency and protein molecular weight also showed the similar trends with human (S2 Table). We do not know whether the similar amino acid usage trend between human and zebra finch is related to the distinctive neural and behavioral traits, but it is worth studying in depth.

There was no significant difference in the average use of 20 amino acids among zebra finch (*Taeniopygia guttata*), chicken (*Gallus gallus*) and human (*Homo sapiens*). The trends of the various amino acid usage frequency were generally consistent.

## Supporting information

S1 Table Comparison of amino acid usage frequency among *Taeniopygia guttata*, *Gallus gallus and Homo sapiens*. (DOCX)

S2 Table Factors affecting amino acid usage in *Taeniopygia guttata*, *Gallus gallus and Homo sapiens*. (DOCX)
