# Supplementary material for: Base composition is the primary factor responsible for the variation of amino acid usage in zebra finch (Taeniopygia guttata)
Source: PLoS One. 2018 Dec 5;13(12):e0204796. doi: 10.1371/journal.pone.0204796 (PMC6281210; doi:10.1371/journal.pone.0204796)
Supplement: S1 Table — (DOCX) [file pone.0204796.s001.docx]

| AA | Codon | GC- rich | AT- rich | Aromatic | *Taeniopygia gutttata* | *Gallus gallus* | *Homo sapiens* |
| --- | --- | --- | --- | --- | --- | --- | --- |
| Ala | GCN | ＋ |  |  | 0.068797 | 0.0720 | 0.0693 |
| Arg | CGN; AG(AG) | ＋ |  |  | 0.061393 | 0.0572 | 0.0567 |
| Asp | GA(TC) |  |  |  | 0.04119 | 0.0485 | 0.0361 |
| Asn | AA(TC) |  | ＋ |  | 0.03488 | 0.0371 | 0.0469 |
| Cys | TG(TC) |  |  |  | 0.02748 | 0.0229 | 0.0232 |
| Gln | CA(AG) |  |  |  | 0.04576 | 0.0459 | 0.0465 |
| Glu | GA(AG) |  |  |  | 0.06185 | 0.0708 | 0.0686 |
| Gly | GGN | ＋ |  |  | 0.06139 | 0.0625 | 0.066 |
| His | CA(TC) |  |  |  | 0.02513 | 0.0253 | 0.026 |
| Ile | AT(TCA) |  | ＋ |  | 0.04514 | 0.0464 | 0.0443 |
| Leu | CTN, TT(AG) |  | ＋ |  | 0.09804 | 0.0972 | 0.1002 |
| Lys | AA(AG) |  | ＋ |  | 0.05973 | 0.0610 | 0.0563 |
| Met | ATG |  |  |  | 0.03142 | 0.0253 | 0.022 |
| Phe | TTT, TTC |  | ＋ | ＋ | 0.03733 | 0.0387 | 0.0379 |
| Pro | CCN | ＋ |  |  | 0.05805 | 0.0548 | 0.0611 |
| Ser | TCN; AG(TC) |  |  |  | 0.08660 | 0.0785 | 0.0811 |
| Thr | ACN |  |  |  | 0.05566 | 0.0520 | 0.0532 |
| Trp | TGG | ＋ |  | ＋ | 0.01722 | 0.0126 | 0.0132 |
| Tyr | TA(TC) |  | ＋ | ＋ | 0.02598 | 0.0292 | 0.0275 |
| Val | GTN |  |  |  | 0.05698 | 0.0627 | 0.0607 |

S1 Table Comparison of amino acid usage frequency among *Taeniopygia gutttata, Gallus gallus* and *Homo sapiens*
