# Supplementary material for: Base composition is the primary factor responsible for the variation of amino acid usage in zebra finch (Taeniopygia guttata)
Source: PLoS One. 2018 Dec 5;13(12):e0204796. doi: 10.1371/journal.pone.0204796 (PMC6281210; doi:10.1371/journal.pone.0204796)
Supplement: S2 Table — (DOCX) [file pone.0204796.s002.docx]

S2 Table Comparison of the factors affecting the amino acid usage among *Taeniopygia gutttata*, *Gallus gallus* and *Homo sapiens*

| species | *Gallus gallus* | *Homo sapiens* | *Taeniopygia gutttata* |
| --- | --- | --- | --- |
| Relationship of tRNA gene copy numbers and amino acid usage | r = 0.6215, p < 0.0001 | r =0.56, p =0.01 | r = 0.478, p = 0.038 |
| Correspondence analysis | The first three axes of COA represented 17.8%, 15.2%, and 8.6% of the total variability,respectively. | The first three axes of COA represented 20.4%, 14.7%, and 9.9% of the total variability, respectively. | The first three axes of COA represented 17.34%, 14.68%, and 8.5% of the total variability, respectively. |
| Contributors of the first major axis | Axis 1 is strongly correlated with the GRAVY score of proteins (r = 0.7341, p<0.0001), and strongly correlated with the Aromo score of proteins (r = 0.5519, p < 0.0001). | Axis 1 is strongly correlated with the GC1, GC2 (axis 1 vs. GCcds, r = -0.69, p < 0.0001; axis 1 vs. GC2, r = - 0.92, p < 0.0001) | Axis 1 is strongly correlated with the GCcds, GC2 (axis 1 vs. GCcds, r = 0.543, p < 0.0001; axis 1 vs. GC2, r = 0.887, p < 0.0001). |
| Contributors of the second major axis | Axis 2 is correlated with the GC1 (r = 0.4509, P< 0.0001), GC2(r = 0.7782, p < 0.0001), and GC cds (r = 0.4608, p< 0.0001) . | axis 2 strongly correlated with the GRAVY score of proteins (r = -0.84, p < 0.0001) | axis 2 strongly correlated with the GRAVY score of proteins (r = 0.732, *p* < 0.0001), and Aromo score of proteins (r = 0.689, p < 0.0001) |
| Effect of isochore structure | Except for Gln and Leu, the RAAU for other amino acids in GC rich isochore significantly differ from that in GC poor isochore. | axis 1 vs. estimator, r = -0.41, p < 0.0001 | axis 1 vs. estimator, r = 0.198, p < 0.0001; axis 2 vs. estimator, r = - 0.16, p < 0.0001 |
| Effect of second structure | Axis 1 correlated with the amount of alpha  helix significantly (r = -0.4440, p< 0.0001，weakly correlated  with the amount of extended strand (r = 0.1465, p< 0.0001), and the amount of random coil (r = 0.0765, p< 0.0001). | Extended strand correlated with axis 2 (r = -0.22, p < 0.0001); Alpha helix and Random coil in each protein strongly  correlated with axis 3 (r = -0.41, p < 0.001; r = 0.48, p < 0.001) | Alpha helix is negatively correlated with axis 1(r= -0.5805, p< 0.0001), but positively correlated with axis 2(r= 0.3133, p< 0.0001). Extend strand is positively correlated with axis 1(r= 0.1553, p< 0.0001) and axis 2 (r= 0.3555, p< 0.0001), but negatively correlated with axis 4 (r= -0.3546, p< 0.0001). Random coil positively correlated with axis 1(r= 0.6012, p<0.0001), and negatively correlated with axis 2 (r= -0.6151,p< 0.0001) |
| Effect of Cys frequency | N | Cys frequency is positively correlated with axis 3 (r= 0.78, p< 0.0001) | Cys frequency is positively correlated with axis 1(r= 0.45, p< 0.0001) and axis 3 (r= 0.5943, p< 0.0001) |
